# Structural Design Principles of Complex Bird Songs: A Network-Based Approach

**DOI:** 10.1371/journal.pone.0044436

**Published:** 2012-09-24

**Authors:** Kazutoshi Sasahara, Martin L. Cody, David Cohen, Charles E. Taylor

**Affiliations:** 1 Graduate School of Information Science, Nagoya University, Nagoya, Aichi, Japan; 2 Department of Ecology and Evolutionary Biology, University of California Los Angeles, Los Angeles, California, United States of America; 3 Department of Electrical Engineering, University of California Los Angeles, Los Angeles, California, United States of America; 4 Department of Electrical and Computer Engineering, Carnegie Mellon University, Moffett Field, California, United States of America; University of Texas at San Antonio, United States of America

## Abstract

Bird songs are acoustic communication signals primarily used in male-male aggression and in male-female attraction. These are often monotonous patterns composed of a few phrases, yet some birds have extremely complex songs with a large phrase repertoire, organized in non-random fashion with discernible patterns. Since structure is typically associated with function, the structures of complex bird songs provide important clues to the evolution of animal communication systems. Here we propose an efficient network-based approach to explore structural design principles of complex bird songs, in which the song networks–transition relationships among different phrases and the related structural measures–are employed. We demonstrate how this approach works with an example using California Thrasher songs, which are sequences of highly varied phrases delivered in succession over several minutes. These songs display two distinct features: a large phrase repertoire with a ‘small-world’ architecture, in which subsets of phrases are highly grouped and linked with a short average path length; and a balanced transition diversity amongst phrases, in which deterministic and non-deterministic transition patterns are moderately mixed. We explore the robustness of this approach with variations in sample size and the amount of noise. Our approach enables a more quantitative study of global and local structural properties of complex bird songs than has been possible to date.

## Introduction

Most passerine birds sing to guard or advertise their territory, alternatively to repel trespassers and attract potential mates [1]. Their songs vary greatly in complexity. Some species, like White-crowned Sparrow (*Zonotrichia leucophrys*) and Zebra Finch (*Taeniopygia guttata* ) sing songs composed of a few monotonous notes and phrases repeated in a fixed sequence [2], [3]. Some others, like American Redstart (*Setophaga americana*) and Bengalese Finch (*Lonchura striata* var. *domestica*), sing more variable songs in which a dozen syllables or phrases are used in different contexts [4], [5]. Still others, notably birds in the family Mimidae such as Northern Mockingbird (*Mimus polyglottos*) and Brown Thrasher (*Toxostoma rufum*), have long complex songs comprising hundreds or even thousands of different syllables or phrases, some of which mimic other species [6].

It is unclear why bird songs should sometimes be so complex. One possibility is that complex songs may reflect a male's age and/or quality, and reliably represent the quality or fitness of the male who sings the song. Another hypothesis is that females inherently prefer complex songs over simple ones, and hence males with complex songs are in some way more attractive to them as mates. These are not mutually exclusive, so that complex songs may confer advantage on the singer both in male-male competition for territory, and in male-female attraction enhancing mating potentials (the so-called ‘dual function hypothesis’ [1]). Although these explanations are intuitively appealing, there are no conclusive experimental results in their support; some positive evidence exists for each in some species, but it is countered by negative indications in other species [7], [8]. In any event, it is clear that improved quantification and analysis of song complexity is necessary for elucidation of its role in avian communication.

Most earlier studies of song complexity have focused on simple parameters such as repertoire size (the number of different syllable-, phrase-, or song-types) and song versatility (the switching rate between song components), rather than on song structure measured by the organization of its components [9], [10]. Repertoire size has been used frequently in comparative studies of songs amongst individuals and species, because it is simple to use and intuitively understood. Simple song metrics, however, are ineffective in characterizing complex songs with large repertoires and, further, the determination of repertoire size is sometimes very difficult to accurately ascertain in large-repertoire species. Though there have been numerous studies that measured repertoire size [11] and that approached song structure using simple statistics and models [12]–[16], the structural properties of complex songs remain largely unexplored. One emerging property is that different phrases are often arranged in sequences that are neither uniform nor random, but possess some intermediate and discernible pattern.

In this paper we propose an efficient network-based approach that quantifies transition relationships among phrases in two different ways, using complex network theory. We analyze songs of the California Thrasher (*Toxostoma redivivum*) as an exemplar of how a network-based approach may capture some essential characteristics of complex bird songs and complement conventional metrics such as repertoire size. We then discuss some possible applications of the network-based approach relevant to an improved understanding of the evolution of animal communication systems.

## Methods

### Song recording

The California Thrasher is a large, ground-foraging passerine found in coastal and foothill chaparral from northern California to northern Baja California. Like the congeneric Brown Thrasher, it has a large repertoire incorporated into long, complex songs consisting of diverse and distinct patterned components, which we term ‘phrases’, variously delivered in a nearly continuous string or sequence for several minutes and punctuated with intermittent brief pauses of a few seconds duration [17] (see Figure S1 for the acoustic features of phrases). A single male may sing several hundred distinguishable phrases within a song bout a few minutes long.

We recorded spontaneous singing of a single male California Thrasher from his territory in foothill chaparral vegetation in Amador County, California, during a single morning on March 21, 2009. Recordings were made along the Comanche Parkway, at approximately 38 deg 15 min 17.95 sec N, 120 deg 53 min 5.43 sec W, 168 m elevation. No birds were touched or in any way manipulated. By limiting our song data to a particular bird and a narrow time slot we hoped to minimize factors that may generate variations in phrase type and phrase usage within the broader population and over time (e.g., daily, seasonal, and age-related changes). We expect to study these variations with our methodology once its utility is established; indeed, that is the objective of the present paper, which we feel is best met by minimizing other variations beyond a target individual singing on territory during a single morning. Different song bouts were recorded in separate WAV files (16-bit, mono, 44.1 kHz sampling rate) using a Marantz PMD 670 with a Telinga parabolic reflector and Sennheiser omnidirectional microphone. The song data were accumulated in 7 recording sessions over a 1.5 h period and total 20 min in overall duration.

### Classification of phrase types

**Figure 1:**
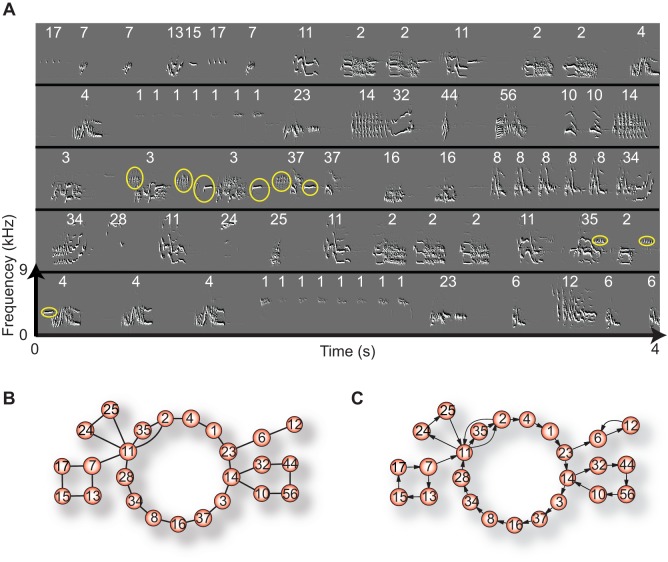
Song fragment from a California Thrasher recording. A. Sound spectrogram of part of a song. Phrases are labeled with their ID numbers. Yellow circles denote background singing of other birds. These are eliminated from analysis. B–C. Song undirected and directed networks constructed from the song fragment shown in A. Nodes represent phrases and edges represent transitions between them, directed or not; self-transitions are omitted.

We computed the sound spectrograms of all the songs using Sound Analysis Pro (http://soundanalysispro.com) with a 9.27 ms FFT data window and a 2.0 ms advancing window. This protocol provided sequences of a total of 2,897 phrases punctuated by periods of silence. Figure 1A shows the sound spectrogram of a 20 sec song fragment in which 71 phrases (of 25 different types) are depicted (File S1). Some of the phrases were always repeated immediately, while others always occurred singly (see Figure S2 for the nature of phrase repetitions). Each phrase was categorized into one of 182 types based on both visual examination of their sound spectrograms and auditory recognition of the recorded sound, and all recognized phrases were classified with reference to a catalog of standards. Each phrase was encoded with an ID number, and songs could then be represented as symbol or number sequences (see Table S1 for the complete catalog).

In order to verify that our classification was objective, we trained a support vector machine (SVM) [18] to classify the phrase categories that been initially chosen subjectively. This was done by training SVMs on samples of phrases that had been chosen by a supervisor to recognize the different phrase types, and then evaluating their ability to classify the remaining phrases that had not been part of the training set. The average percent correct classification for the 25 test phrase types was 97.5% and for the ‘other’ category was 92.0% (see Information S1). Therefore we conclude that our classification of phrase types, while initially subjective, was objectively confirmed.

### Song network analysis

The song network analysis that we propose focuses on transition relationships among the different phrases. First of all, transitions between successive phrases are analyzed for each adjacent phrase pair along the song sequences; n.b. instances of repetition of the same phrase (‘self-transitions’) are omitted because standard network measures, discussed below, are typically defined for networks without self-transitions. Then, based on observed transitions, we construct a ‘song network’, which may be either an undirected or a directed graph, in which nodes represent different phrases and edges represent transitions between them. We term these ‘song undirected networks (SUNs)’ and ‘song directed networks (SDNs)’. Figure 1B and 1C are the examples of SUN and SDN constructed from the song fragment shown in Figure 1A. Thus phrase sequences may be represented by both types of network, which can then be characterized by a few global and local network measures as described below. With this coarse-grained procedure, we may lose some details of the song properties, but we encompass basic song complexity within the aegis of established network theory.

#### Song undirected networks and measures

In the SUNs, nodes represent phrases, and undirected edges represent transitions or links between them. Bi-directional or reversible transitions (e.g., phrase ID#s 6→12, 12→6 in Figure 1A) are not distinguished, so both occur in the edge joining phrase ID#s 6 and 12. The SUN is the minimal representation of the connection topology among different phrases, and can be characterized by three network measures: average path length, clustering coefficient, and degree distribution [19].

Average path length, *L*, is defined as the average minimum number of connections to be crossed from any arbitrary phrase to any other. *L* measures the overall navigability in the song network; as *L* becomes large, a longer series of transitions, involving more steps, is required for any phrase to reach another. Such a situation might be found in a stereotypic song composed of many different phrases sung in a fixed sequence. On the other hand, if *L* is small, the opposite happens, such as in more versatile songs that can loop back to previous phrases through shorter and alternative intermediate sequences.

The clustering coefficient, *C*, is the overall tendency of different phrases to form groups that are highly likely to co-occur in a song sequence, computed as follows:
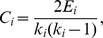


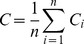



For each phrase *i*, there are 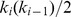 possible links within the set of 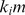 phrases linked to phrase *i*. *E_i_* is the observed number of such links for phrase *i*, and thus *C_i_* measures the proportion of possible links that is actually observed. *C* averages *C_i_* values over all phrases *m*. Song networks with more and more connected phrase groups in them will have higher *C* values. If there is no grouping of phrases then *C* = 0; if every pair of phrases is fully connected then *C* = 1.

Degree distribution is the distribution over *k* of *P*(*k*), the probability that a phrase has *k* connections or transitions. Its shape is helpful in distinguishing between different classes of networks; for example, a bell-shaped degree distribution is seen in a random network, and indicates that there are no highly connected phrases known as ‘hubs’. In contrast, a degree distribution with a long tail to the right (at high *k* values) indicates that there are hubs in the song network, unlike random networks.

#### Song directed networks and transition motifs

Complex networks in nature commonly contain recurring patterns of inter-connections, sometimes termed ‘network motifs’ [20]. Song directed networks may also display such small-scale structural patterns; to quantify these, we introduce five types of ‘transition motifs’ defined by the combination of incoming (in-degree) and outgoing (out-degree) edges. These motifs are ‘One-way’ (one-to-one), ‘Bottleneck’ (many-to-one), ‘Branch’ (one-to-many), ‘Hourglass’ (many-to-many), and ‘Margin’ (either no in-degree or no out-degree). One-way and Bottleneck motifs are deterministic, in that one or more phrases transit to a specific phrase; Branch and Hourglass motifs are non-deterministic, in that transitions through a specific phrase may transit to one of several different phrases; Margin motifs mark the beginnings or ends of song sequences.

Local structural properties of complex bird songs can be characterized by the transition motifs in the song directed network. For example, a highly versatile or randomly organized song may be mostly occupied by nondeterministic transition motifs, whereas a stereotypic song may consist of mainly deterministic transition motifs (both along with Margins). In contrast to these extremes, a complex song can exhibit moderately mixed transitions, characterized by a particular ratio of deterministic and nondeterministic motifs.

#### Random networks for comparison

The structure of observed song networks can be compared to random networks having the same overall topology. We generate ‘random undirected networks (RUNs)’ and ‘random directed networks (RDNs)’ with the same number of nodes *n*, and the same average degree 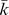 (average number of edges per node) as the SUN and SDN, respectively. These random networks are constructed in a manner similar to the Erdös-Rényi model [21]. In the case of RUN, an undirected edge is placed with probability *p* at each of *n* nodes; if the resulting RUN has exactly the same 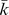 as SUN, it is retained; otherwise, the procedure is repeated until the criterion is met. In the case of RDN, one of the following directed edges is placed at each of *n* nodes with equal probability *p*: in-degree, out-degree, and bi-degree; if the resulting RDN has exactly the same 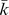 as SDN, it is retained; otherwise, the procedure is repeated as before.

Random networks provide baseline data for comparison with observed song networks. If the measures of song networks (*L*, *C*, and *P*(*k*)) are similar to those of the RUN or RDN, the observed songs may be just random assemblies of phrases; if, however, the properties of actual or observed song networks are quite different from RUN and RDN, then other design principles must underlie their construction. In drawing such comparisons, it should be recognized that Erdös-Rényi-type random undirected networks, in general, have small *L*, small *C*, and *P*(*k*) distributed as a bell-shaped curve [19].

## Results

We demonstrate how this approach works with California Thrasher songs, and evaluate the robustness of the approach with variation in sample size and errors in phrase identification.

### Song undirected network analysis

We constructed a song undirected network (SUN), using the entire set of phrase sequences. All networks were characterized with three network measures: average path length *L*, clustering coefficient *C*, and degree distribution *P*(*k*), as described above. We compared the observed song network with 1,000 corresponding random undirected networks (RUNs) that had the same number of nodes *n* and average degree 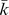 as the SUN (*n* = 182 and 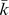 = 6.4, respectively), by calculating 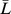 and 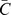, and *P*(*k*) for the RUNs.

**Figure 2:**
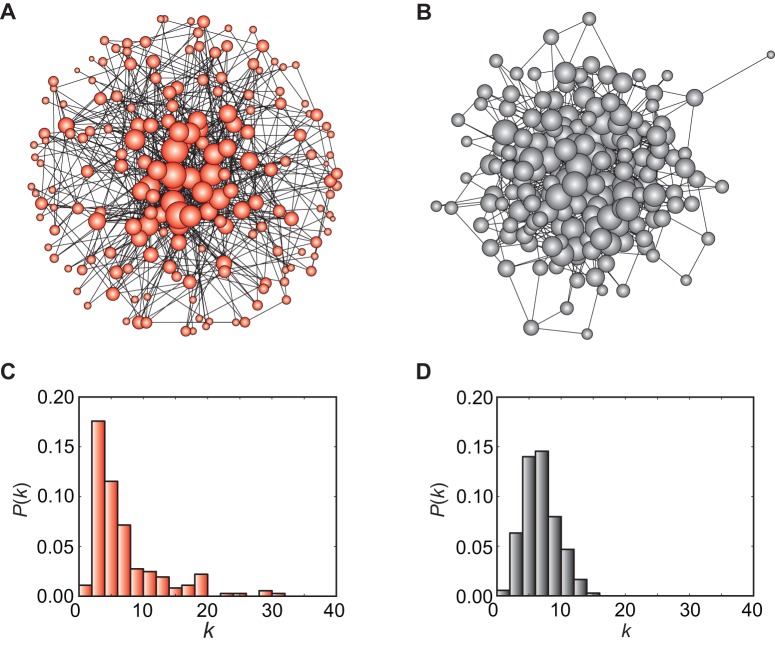
Global structural properties of song and random undirected networks. A. Song undirected network (SUN) constructed from all the recordings. B. Random undirected network (RUN) that has exactly the same *n* and 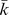 as the SUN. The estimated *L* and *C* in the SUN are 3.15 and 0.21, respectively. The estimated *L*_rand_ and *C*_rand_ being average values computed from 1,000 different RUNs are 2.98 and 0.03, respectively. Nodes are drawn with sizes proportional to their degree *k*. Degree distribution of the SUN (C) and RUN (D).

Figures 2A and 2B are the observed SUN and a sample RUN. Both networks have small but similar values for *L* (*L* = 3.15 for the SUN, 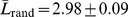 for the RUN; *Z* = 1.9, NS), indicating that in the SUN any phrase can reach any other in just a few transitions–about three degrees of separation, despite a phrase repertoire size *n* = 182. However, the SUN has a value of *C* that is significantly larger than that of the RUN (0.21 vs 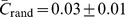 for the RUN; *Z* = 18.0, *P*<0.0001), indicating that there are many more tightly-clustered phrase groups in real songs than in the randomly generated networks. Here *Z*-score is computed as 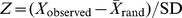, where *X* is either *L* or *C*. Networks like the observed song network, with small *L* and larger *C* relative to random networks, are termed ‘small-world networks’ [22]. Small-world networks are widespread in biological, social, technological, and information systems (including human language), and their collective dynamics have been much studied [22]–[25]. Small-world-ness is typically measured by 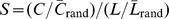, with the property that if *S*>1 the network is regarded as small-world [26]. In this instance, *S* = 6.62.

The third network metric, *P*(*k*), reveals that the SUN and the RUN are quite different types of networks: the *P*(*k*) of the SUN has a long tail to the right over higher *k* values, whereas that of the RUN is approximately a bell-shaped curve. Thus the structure of the California Thrasher song is characterized by phrases grouped into distinct hubs, to an extent not seen in the random networks.

The comparison of the SUN with 1,000 different RUNs suggests that the California Thrasher song is indeed complex but not at all random; rather, it is structured with a small-world topology, which is a property also possessed by human languages [27]. The distinct combination of small *L*, large *C*, and a non-bell-shaped *P*(*k*) is unlikely to emerge if phrases were randomly distributed in the songs. In this thrasher, songs appear to be governed by principles consistent with a small-world network organization over a large phrase repertoire.

### Song directed network analysis

**Figure 3:**
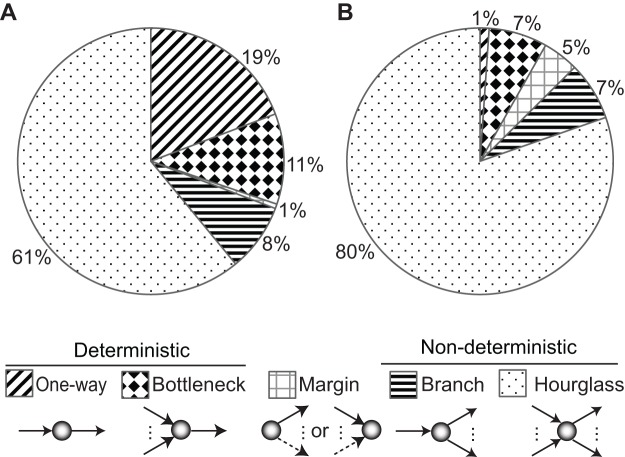
Local structural properties of song and random directed networks. A. Configuration of transition motifs in the song directed network (SDN). B. Same as in the comparable random directed network (RDN) that has exactly the same measures of *n* and 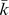 as the SDN, estimated from 1,000 different RDNs. Both networks are characterized by five types of transition motifs indicated: One-way, Bottleneck, Branch, Hourglass, and Margin, with proportions given in the diagrams.

The song directed network (SDN) is characterized with the five types of transition motifs described above and shown in Figure 3. For the purposes of comparison, we constructed 1,000 corresponding random directed networks (RDNs) with the same values of *n* (182) and 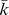 (7.5) as in the observed SDN. In the SDN and the RDNs, the average path length is comparable (*L* = 4.17 for the SDN and 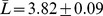 for the RUN); again, it suggests that the bird can switch one phrase type to any others with a small number of steps even in the song directed network.

Figures 3A and 3B show pie charts of the proportion of transition motifs contained in the SDN and in the RDN, respectively, with average values shown for the latter. It is evident from this figure that the observed SDN has different proportions of transition motifs compared with the RDN (Chi-square test with four degrees of freedom; *P*<0.0001). Denoting the number of appearances in the real networks as *N*_SDN_ and in the random network as *N*_SDN_, we computed *Z*-scores for each transition motif as above. The SDN contains a larger proportion of deterministic transition motifs: One-way ((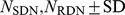)  =  (35,2±1), *Z* = 33.0, *P*<0.0001) and Bottleneck ((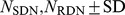)  =  (20,13±2), *Z* = 2.3, *P*<0.05). At the same time, the RDNs contain many more non-deterministic transition motifs: Hourglass ((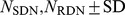)  =  (111,146±4), *Z* = −8.8, *P*<0.0001), and more Margin ((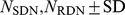)  =  (1,8±3), *Z* = −2.3, *P*<0.05). We found no statistically significant difference in Branch motifs ((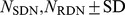)  =  (15,13±3), *Z* = 0.17, NS). Together, our results indicate that the deterministic and non-deterministic transition motifs are moderately mixed in the actual song network, compared to the heavily non-deterministic nature of the random directed network.

**Figure 4:**
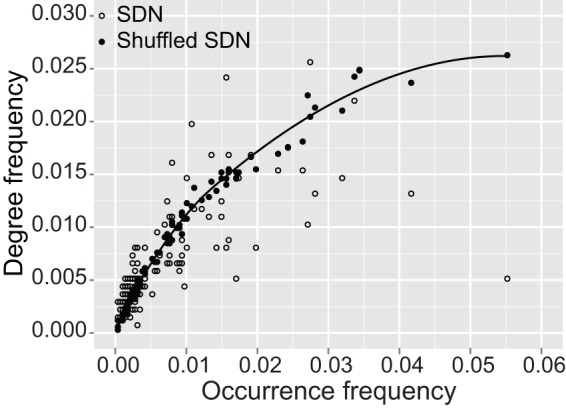
Correlation between the occurrence frequency and the degree frequency of phrases. The song directed networks (SDNs) are constructed from real songs and from shuffled songs. The line shows a locally weighted scatter plot smoothing for the shuffled SDN.

In addition, in Figure 4, we illustrate another feature of song structure derived from a comparison of real songs with ‘shuffled’ songs. Shuffled songs were generated by 1,000 repeated position switches of randomly-selected phrase pairs from the original song sequences; thus, the original frequency distribution of phrases is maintained but the transition contexts are altered. The figure compares the occurrence frequency, obtained by dividing the number of each phrase by the total number of phrases, with the degree frequency, the degree of each phrase divided by the total degree. If transitions between phrases were random, common phrases would gain more connections after the shuffling, i.e. attain a higher degree, because they have more chances to become involved in novel transitions. The observed SDN follows this expectation for less popular phrases, but with increasing occurrence frequency the degree frequency falls increasingly below this expectation. In particular, several of the most popular phrases are characterized by only moderate degree frequencies, indicating some stereotypy in their transitions. This indicates that both random and non-random processes play important roles in creating the diversity of phrase-to-phrase transitions in complex bird songs.

### Sample size dependence

The structural properties of a song networks described above might conceivably be dependent on sample size, the song length, or total number of phrases recorded. To explore this possibility and to test for the robustness of the structural properties we have described, we constructed SUNs from songs of different sequence lengths, and computed the corresponding network measures.

**Figure 5:**
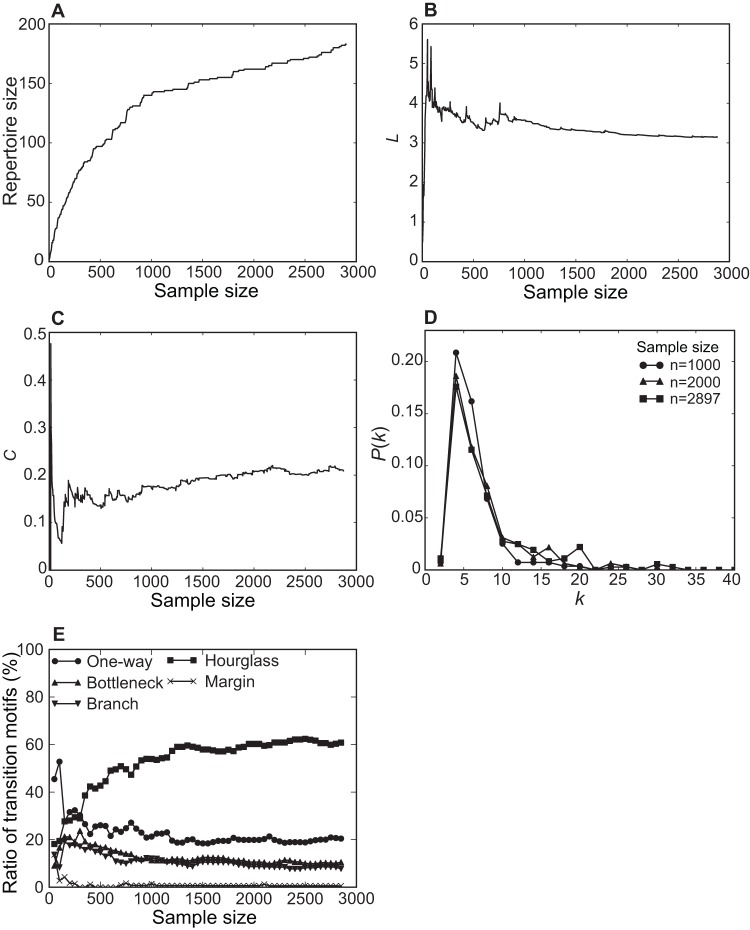
Sample size dependence of song network analysis. A. The cumulative plot of phrase repertoire size (number of different phrases) as a function of sample size (total number of phrases in song). B–C. Average path length *L* and clustering coefficient *C* as a function of sample size. D. Degree distribution *P*(*k*) at *n* = 1,000, 2,000 and 2,897. E. The ratio of five transition motifs as a function of sample size.

As expected, the number of different phrases observed (repertoire size) increased when a larger number of phrases (longer song) were used (see Figure 5A), though at a decreasing rate with no evident asymptote. Thus a limit to repertoire size is not discernable, and is undoubtedly much greater than the number we observed (182 different phrases). There are two important consequences to this. First, unlike simple songs, the complex songs we recorded, even from just one individual over a short time interval, cannot be described by a single measure like repertoire size. Second, because the simple measures of complexity are sensitive to song length, there is an evident need for scale-free measures of song complexity.

For the network measures, the outcome is different, because of far greater sample size independence; *L* and *C* already approach an asymptote at *n* = 2,000 (see Figure 5B and 5C), and *P*(*k*) exhibits almost the same distribution at *n* = 2,000 and *n* = 2,897 (see Figure 5D). We take this convergence of *L*, *C* and *P*(*k*) as evidence that the three network measures were successfully estimated by our samples, and are more robust indicators of song complexity than is repertoire size. The proportions of transition motifs were similarly independent of sample sizes. We constructed SDNs with different sample sizes and measured variation in the proportions of transition motifs. Figure 5E illustrates how the composition of transition motifs changes as a function of sample size. In this figure the distribution of transition motifs is stabilized beyond about 50% of our maximum sample size. The proportions we observed of stochastic vs. deterministic transitions are not dependent on sample size, but are rather real properties of song structure.

Both types of analyses support the conclusion that, while phrase repertoire size does increase with longer observation periods, the same is not true for the network-based measures, which stabilize at samples sizes considerably less than our maximum. In view of this stability, it is apparent that some 20 minutes of song recording was sufficient for characterizing California Thrasher song complexity with scale-invariant network measures.

### Noise rate dependence

**Figure 6:**
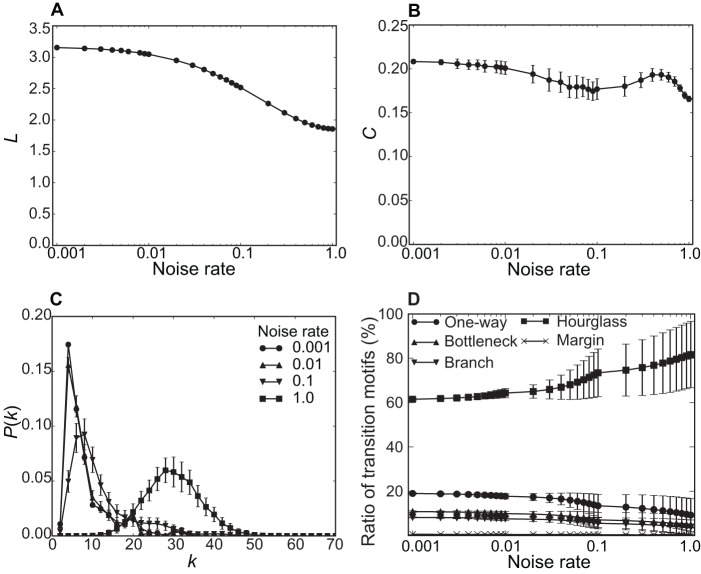
Noise rate dependence of song network analysis. A–B. Average path length *L* and clustering coefficient *C* as a function of noise rate. C. Degree distribution *P*(*k*) at different noise rates. D. The ratio of five transition motifs as a function of noise rate. Each data point is the average and SD of 100 noise-induced experiments with different random seeds.

Measurement of song complexity might well be affected by misclassification of song phrases. To examine this possibility, we performed a series of experiments, in which selected phrases in the original song were replaced at random with other phrases. This procedure imitates phrase misclassification. The network measures used above were then recomputed as a function of noise rates–the ratio of the number of misclassifications to the total number of phrases (see Figures 6A–D). In these figures, each data point is the average and standard deviation of 100 noise-induced experiments with different random seeds.

These results suggest that a modest amount of noise (i.e., several tens of misclassifications out of 2,897 phrases) makes little difference for the various network measures. Beyond modest noise rates, however, the network measures begin to change substantially; both *L* and *C* decrease and the peak position of *k* shifts toward higher values. In other words, misclassification generates denser phrase connectivity, reflected by a higher proportion of Hourglass motifs. At still greater noise rates (>10%, i.e., a few hundred misclassifications), we get networks that are no longer comparable with the original song network. Thus, phrase misclassification at the level of a few percent would make little difference to our characterization of song structure.

### Comparison of song networks between different individuals

Here we analyze song data from two additional individual thrashers in the Santa Monica Mountains, California, and compare song structure in these individuals to that we have analyzed above (referred to as Mar2009). The additional recordings (referred to as Feb2012-1 and Feb2012-2, respectively) were made on February 8–10, 2012, from along the Topanga Lookout Road, at approximately 34 deg 4 min 57.98 sec N, 118 deg 38 min 35.60 sec W, 740 m elevation, with the same recording equipment and conditions. The song data of Feb2012-1 and Feb2012-2 comprise sequences of 658 and 1,316 phrases, respectively; phrase type classification and song network analysis are performed in the same manner as described above.

The result of song undirected network analysis is summarized in Table 1. All the birds exhibited positive values of small-world-ness (*S*) and non bell-shaped degree distribution *P*(*k*) (see Figure S3), indicating that the songs of these California Thrashers share a small-world property, despite large individual differences in the sizes of their phrase repertoires and their specific phrase types. Furthermore, Table 2 shows that Feb2012-1 and Feb2012-2 have similar composition of transition motifs in song directed networks (i.e., a large portion of One-way motifs), and that all the birds possess diverse transition motifs with a different balance of corresponding random networks (Chi-square test with four degree of freedom; *P*<0.0001. See also Information S1).

**Table 1:** Comparison of song undirected networks between different individuals.

SUN	Nodes	Edges	*L*	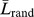	*C*	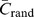	*S*
Mar2009	182	580	3.15	2.98±0.09	0.21	0.03±0.01	6.62
Feb2012-1	57	149	2.81	2.58±0.03	0.37	0.09±0.02	3.68
Feb2012-2	120	257	6.61	3.44±0.04	0.31	0.03±0.01	4.86

(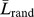 and 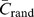 are computed from 1,000 corresponding RUNs.).

**Table 2:** Comparison of song directed networks between different individuals.

SDN	One-way	Branch	Margin	Bottleneck	Hourglass
Mar2009	0.19 (0.01)	0.08 (0.07)	0.01 (0.04)	0.11 (0.07)	0.61 (0.81)
Feb2012-1	0.37 (0.02)	0.07 (0.11)	0.00 (0.07)	0.07 (0.11)	0.49 (0.70)
Feb2012-2	0.38 (0.04)	0.10 (0.15)	0.01 (0.15)	0.08 (0.15)	0.43 (0.51)

(The average values computed from 1,000 corresponding RDNs).

With analysis of these additional song data from different individuals, our methodology gains further support of its effectiveness to quantify and compare the non-random structures of complex bird songs. We note that individual differences of song structure observed here may result from seasonal and/or geographic factors, and planned research will clarify these influences by obtaining a more systematic recording across seasons and regions.

## Discussion

We have described a network-based approach to explore the structural properties of complex bird songs–both global and local features. Our approach helps to elucidate the design principles of complex bird songs in a way that is difficult with conventional methods only. It enabled us to identify distinct structural features of California Thrasher songs, such as a large phrase repertoire that is organized as a classical ‘small-world network’, with a balance of deterministic and non-deterministic transition patterns between adjacent phrases, although the generality of the results must be tested in many more California Thrashers, at other times and places, and in other species recognized for their complex songs, such as Nightingales, Northern Mockingbirds, and Brown Thrashers. As far as we know, this is the first attempt at utilizing a network-based approach to the structural design principles of complex bird songs, although similar approaches have been taken in other aspects of animal behavior studies, mostly in the context of social network analysis [28].

The pros and cons of our approach may be assessed in comparison with conventional methods: repertoire size, versatility, information entropy, and Markov models. As stated before, repertoire size and versatility have been commonly used as measures of song complexity, promoted undoubtedly by their simplicity of application. In large repertoire songs, however, these measures are not easily estimated, as seen in Figure 5A; further, they are not necessarily good indicators of song complexity, since a large phrase repertoire organized into one fixed sequence could not be deemed especially complex. Information entropy and Markov models can provide more insight into how repertoire is organized. Since both measures are based on a transition matrix describing the probabilities of moving from one component type to another, they require accurate estimates of the occurrence frequencies of transitions among component types, which in turn require large sample sizes. However, to construct song networks (either SUN or SDN), transition probabilities are not necessary; instead, we need to know binary information about whether transitions amongst phrase types occur or not, which can be estimated from a relatively small samples size. There are, of course, some constraints to a network-based approach. Since complex network theory is targeted at networks consisting of a large number of nodes (e.g., *n*>50) with sparse connections between them [22], network measures are of no value for small-size networks (e.g.,*n* = 10 or fewer). In sum, our approach neither supplants nor contradicts conventional methods, but rather supplements them by characterizing complex song structure at adequate levels of abstraction.

We suggest three possible applications of the network-based approach. First, in addition to the conventional measures, this approach can provide tools for quantifying intra- and inter-specific differences in complex songs. By studying these variations we may approach the following sorts of questions: Are all complex bird songs governed by the same structural design principles, or might there be different ones in different species? Second, the ability to learn songs, thought to be absent in non-passerine birds, is assumed typical of oscine passerines [29]. But what aspects of song structure are innate versus learned in oscine passerines? Because a song network is a learned outcome and an individual construction, complex bird songs may provide a generally useful biological model of how a large phrase repertoire develops, how a small-world architecture originates, and how a particular configuration of transition motifs emerges during the learning process. By tracking song development in a controlled environment, it may be possible to quantify song learning as phrase repertoire growth within a song network framework. Third, although some previous studies have produced evidence that song complexity has evolved because of female preference for males with larger repertoires [30]–[33], the structured song network we studied might require another explanation: female preference for more abstract song features, such as combinatorial aspects or transition modes, might be driving the evolution of signal complexity [34]. Whether or not females can respond to such abstract song features could be tested by song playback experiments, in which synthesized songs that control for variation in *L*, *C*, and *P*(*k*), or in the proportions of transition motifs, are played to females whose responses can be measured and studied.

Clearly, past studies of complexity in bird songs that focused exclusively on simple song parameters addressed only a small part of song structure, representing just the ‘tip of the iceberg.’ With the use of methods that are standard in network analysis, a rich set of patterns may be revealed that can further our understanding of the evolution of vocal communication in birds, of animal communication systems in general, and of questions of syntax and even semantics that approach those asked by students of human language. Objective and quantitative analysis, exemplified by network-based approaches such as ours, will be fundamental to this understanding.

## Supporting Information

Figure S1 **Repertoire diversity in the acoustic feature space.**
(EPS)

Figure S2 **Statistical prosperities of repetitions of same phrase.**
(EPS)

Figure S3 **Comparison of degree distributions of different individuals.**
(EPS)

Information S1 **Detailed description of California Thrasher song and of a method of phrase classification.**
(PDF)

Table S1 **Catalog of phrase repertoire.**
(EPS)

File S1 **Audio file of a California Thrasher song.**
(WAV)
