# Supplementary material for: Structural Design Principles of Complex Bird Songs: A Network-Based Approach
Source: PLoS One. 2012 Sep 24;7(9):e44436. doi: 10.1371/journal.pone.0044436 (PMC3454418; doi:10.1371/journal.pone.0044436)

Supporting Information S1
Structural Design Principles of Complex Bird Songs: A Network-Based Approach

Acoustic Diversity of Phrase Repertoire

We recorded spontaneous singing from a California Thrasher recorded in Amador County, California in March 2012 (referred to as Mar2009). Using every representative datum of all the phrase types, we made a phrase repertoire catalog with ID number assigned in descending order of its frequency (1 is the most popular phrase and 182 is the rarest one). From these data we extracted five acoustic features—phrase duration, mean pitch, mean Wiener entropy, mean goodness of pitch, and mean FM—using Sound Analysis Pro (`http://soundanalysispro.com`) [1]. Principal component analysis (PCA) was then applied to the data set in order to reduce its dimensionality. Figure S1 shows repertoire diversity in the acoustic feature space defined by the first two principal components. While there are some distinct phrases with longer duration and higher pitch, most are similar in duration and mean pitch but differ in the other features, as seen in Figure S1. These regularities may reflect morphological constraints on acoustic diversity in a phrase repertoire. The fact that most of the phrases occupy a distinct place, indicates the validity of our phrase categorization, which is further examined with the SVM, described bellow.

Repetitions of Same Phrase

In the song network analysis, the repetitions of the same phrase are omitted because the network measures: average path length, L, clustering coefficient, C, degree distribution, $P(k)$, and transition motifs are computed only for networks without self-loops. Repetitions of same phrase, however, are another important feature in bird song, and thus we analyzed it in our data set. Figure S2A shows a frequency distribution of repetition rates of all the phrases in the data of Mar2009, indicating that about 60% of the phrases do not show up in repetitions, but the others do. Furthermore, Figure S2B shows the number of repetitions as a function of the phrase ID (n.b. 1 is the most popular phrase and 182 is the rarest one). We see that the most common phrase is often repeated, but in variously sized repeats, four up to eight, and that another less popular phrase, ranked 68, repeats nine times. Overall, there seems to be no obvious relationship between the number of repetitions and the popularity of phrase types.

Comparison Between Different Individuals

We describe other statistical properties of two additional California Thrashers that were recorded in the Santa Monica Mountains, California in February 2012 (referred to as Feb2012-1 and Feb2012-2, respectively). Figure S3 shows the degree distributions of these birds together with Mar2009 data. Despite individual differences in the sizes of phrases and their types, all the birds show similar non bell-shaped degree distributions in the SUNs, which are highly unlikely to emerge in the corresponding RUNs.

Additionally, we summarize the statistics of transition motifs in these thrashers. In the song directed network (SDN) of Feb2012-1, the transition motifs are summarized as follows: in One-way, $(N_{\mathrm{SDN}}, N_{\mathrm{RDN}} \pm \mathrm{SD}) = (21, 1 \pm 1)$, $Z = 22.6$, $P < 0.0001$; in Branch, $(N_{\mathrm{SDN}}, N_{\mathrm{RDN}} \pm \mathrm{SD}) = (4, 6 \pm 2)$, $Z = -1.0$, NS; in Margin, $(N_{\mathrm{SDN}}, N_{\mathrm{RDN}} \pm \mathrm{SD}) = (0, 4 \pm 2)$, $Z = -2.2$, $P < 0.05$; in Bottleneck, $(N_{\mathrm{SDN}}, N_{\mathrm{RDN}} \pm \mathrm{SD}) = (4, 6 \pm 2)$, $Z = -1.0$, NS; in Hourglass, $(N_{\mathrm{SDN}}, N_{\mathrm{RDN}} \pm \mathrm{SD}) = (28, 40 \pm 3)$, $Z = -4.2$, $P < 0.0001$. In the SDN of Feb2012-2, the transition motifs are as follows: in One-way, $(N_{\mathrm{SDN}}, N_{\mathrm{RDN}} \pm \mathrm{SD}) = (46, 5 \pm 2)$, $Z = 19.3$, $P < 0.0001$; in Branch $(N_{\mathrm{SDN}}, N_{\mathrm{RDN}} \pm \mathrm{SD}) = (12, 18 \pm 3)$, $Z = -1.6$, NS; in Margin $(N_{\mathrm{SDN}}, N_{\mathrm{RDN}} \pm \mathrm{SD}) = (1, 19 \pm 4)$, $Z = -4.9$, $P < 0.0001$; in Bottleneck $(N_{\mathrm{SDN}}, N_{\mathrm{RDN}} \pm \mathrm{SD}) = (9, 18 \pm 4)$,

$Z = -2.5$, $P < 0.05$; in Hourglass, $(N_{\mathrm{SDN}}, N_{\mathrm{RDN}} \pm \mathrm{SD}) = (52, 61 \pm 4)$, $Z = -2.2$, $P < 0.05$. Here Z-score is measured as $Z = (N_{\mathrm{SDN}} - N_{\mathrm{RDN}})/\mathrm{SD}$, where $\bar{N}_{\mathrm{RDN}}$ is computed from 1000 random directed networks (RDNs). The SDNs of Feb2012-1 and Feb2012-2 have more One-way motifs than the SDN of Mar2009 and the corresponding RDNs.

Evaluation of Phrase Classification with Support Vector Machines (SVMs)

In order to verify that our classification was objective, we trained a support vector machine (SVM) to classify the phrase categories that been initially chosen subjectively. This was done by training SVMs on samples of phrases that had been chosen by a supervisor to recognize the different phrase types, and then evaluating their ability to classify the remaining phrases that had not been part of the training set. Training sets consisted of 15 examples, chosen at random, for each of the 25 most common phrase types. If the SVM could not confirm membership in one of the 25 phrases in the training set, then it assigned the test phrase to an "other" category that consisted of all remaining phrase types. We ran 5 independent replicates of randomly sampled training and testing sets. The rationale for choosing an SVM, a description of the program we used, and the features of the phrases used for the classification are described below. The program and usage notes are available online (`http://taylor0.biology.ucla.edu/al/bioacoustics/`).

SVM Theory Introduction

The Support Vector Machine (SVM) is a binary classification algorithm used widely in the machine learning community [2]. Given training data points $\boldsymbol{x}_i$ with known labels $y_i \in \{+1, -1\}$, the SVM finds the separating hyperplane $\boldsymbol{w}^T\boldsymbol{x} + b = 0$ which maximizes the margin between classes. Intuitively, by maximizing the margin between classes, the classifier is able to generalize to unseen data. Often in practice, classes within data sets cannot be linearly separated. Furthermore, sometimes it is desirable to allow training data to be misclassified in exchange for a classifier that generalizes better to unseen data. The SVM is formulated to allow for misclassified training data in exchange for a larger margin on the correctly classified points. The SVM is formulated as the following optimization problem:

$$\begin{aligned} \mathrm{Min}_{\boldsymbol{w},\xi} \quad & \frac{1}{2}||\boldsymbol{w}||^2 + \gamma \mathbf{1}^T\xi \\ \text{Subject to} \quad y_i(\boldsymbol{w}^T\boldsymbol{x}_i + b) &\geq 1 - \xi_i \\ \xi &\geq 0 \end{aligned}$$

The parameter γ is the weight put on misclassified samples ξ, and represents the trade-off between margin width and training error rate. γ may be tuned using cross-validation.

In practice, data often do not show useful linear structure. A widely used approach for dealing with this is to augment the data and transform them into a higher dimensional inner-product space. Along these dimensions, data that were not previously separable may become separable. Using the Lagrange dual formulation of the SVM, this transformation is done implicitly using the 'kernel trick'. In these experiments, a Gaussian radial kernel function is employed, which implicitly maps input feature vectors to an infinite-dimensional space before the separating hyperplane is found.

$$k(\boldsymbol{x}_i, \boldsymbol{x}_j) = exp\left(-\frac{||\boldsymbol{x}_i - \boldsymbol{x}_j||^2}{2\sigma^2}\right)$$

The variance of the Gaussian kernel, σ^2, is another parameter whose appropriate value depends on the scale of the data, and may be tuned using cross-validation. After applying kernel methods, a function for the separating hyperplane can no longer be practically found. The trained SVM consists of a set of weights α_i that allow classification of any new point $\boldsymbol{x}_{new}$ by calculating:

$$\text{score} = \sum_i \alpha_i y_i k(\boldsymbol{x}_{new}, \boldsymbol{x}_i)$$

For a binary classifier, $y_{new} = \text{sign(score)}$. Many of the weights α_i will be equal to 0, so the classifier does not have to hold all the training data in memory. The training data it does hold are known as support vectors.

The SVM is formulated as a binary classifier, but with bird phrases, the goal is to distinguish between up to dozens of classes. This is achieved by training one-versus-the-rest SVM's for each class. A given test data point is first run through each classifier. The classifier that returns the highest score is then chosen. Data points that do not meet a threshold score for any of the known classes are labeled 'uncertain.' The appropriate threshold is determined through cross-validation. Parameters for the SVM analysis for classification verification above were: number of phrase subdivisions = 4, weight of phrase length $= 0.5, \sigma = 0.5, \gamma = 1.0$, and threshold = 0.7.

Feature Selection

A classifier operates on feature vectors, which must be extracted from the song data. The features selected in this work to classify phrases attempt to concisely summarize the frequency structure over time of the sound signals. The song data are recorded in wav files (16-bit, mono, 44.1 kHz sampling rate). The sound signal is then divided into overlapping segments of 256 samples, where a segment begins every 128 samples. A Hamming window function is then applied and a sliding window discrete Fourier transform is performed to create a sound spectrogram. This contains frequency information from the signal along narrow time slices. The frequencies of the sound spectrogram are then limited to a range of interest. In these experiments, frequency information in the range 1.4 to 6.9 kHz was used.

Within a single phrase, there are changes in the frequency content over time. To capture the distinct sections of frequency characteristics, each phrase is subdivided into a small number of equal length chunks. For each chunk, the average frequency spectrum becomes the final set of representative features. In this work, four subdivisions were used. By doing this, some of the structure information that defines the phrase is lost, but most of the relevant information is concisely summarized. Also, this process changes signals of different length into feature vectors that all have the same length, which is required for the Gaussian radial kernel function. Since this procedure all information about the phrase's absolute length, the final feature vector also includes the weighted log length of the phrase as a separate feature. Using the log length as opposed to the linear length has the advantage that differences in length are considered in proportion to a phrase's absolute size. The weight that is given to a phrase's length information versus its frequency information may be determined through cross-validation.

The SVM classifier program we developed (available for download at `http://taylor0.biology.ucla.edu/al/bioacoustics/`) takes as input a .TextGrid file from Praat (`http://www.fon.hum.uva.nl/praat/`) with the beginning and end times of the phrases to be classified delineated by hand, along with a corresponding WAV file. The program uses a random subset of the data to train an SVM, and can immediately show the performance on withheld data. The program can output a classifier .cl file that can be used to classify later samples from the same species. The program can also output the support vectors and weights of the trained SVM in .csv format, which can be later used in other applications; for example to classify phrases in real time.

References

1. Tchernichovski O, Nottebohm F, Ho C, Pesaran B, Mitra P (2000) A procedure for an automated measurement of song similarity. Animal Behaviour 59: 1167–1176.

2. Cortes C, Vapnik V (1995) Support-vector networks. Machine Learning 20: 273-297.

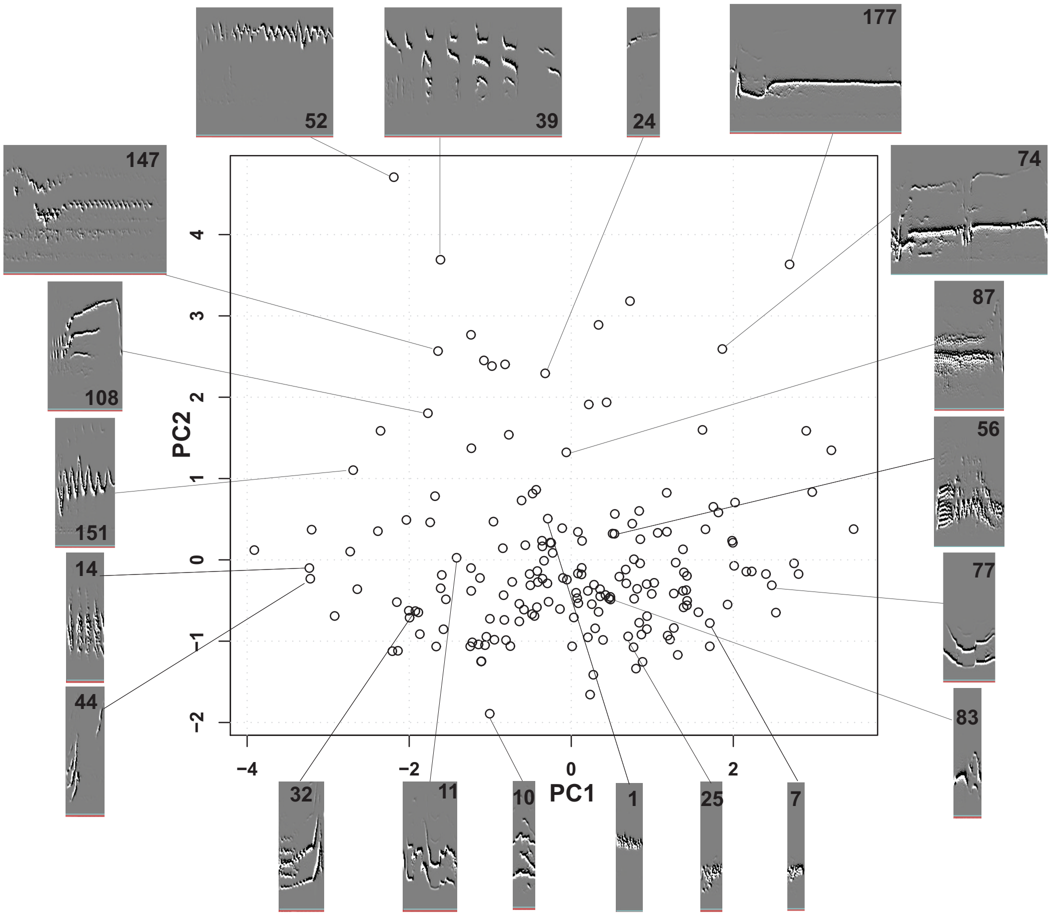

Figure S 1. Repertoire diversity in the acoustic feature space.

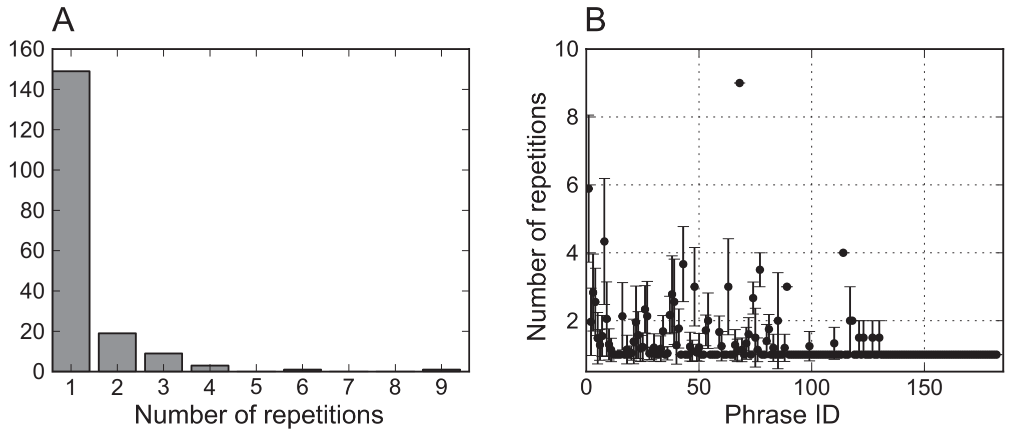

Figure S 2. Statistical prosperities of repetitions of same phrase. A, Frequency distribution of the repetitions of same phrase. B, Correspondence between the number of repetitions and the popularity of phrase types.

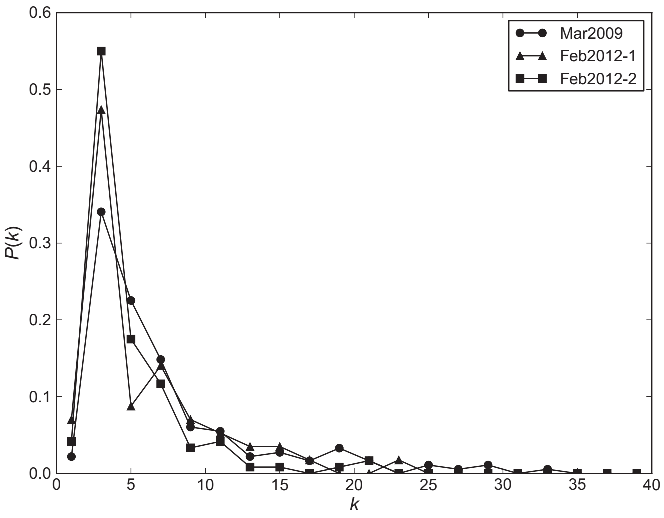

Figure S 3. Comparison of degree distributions of different individuals.

Table S1: Catalog of phrase repertoire

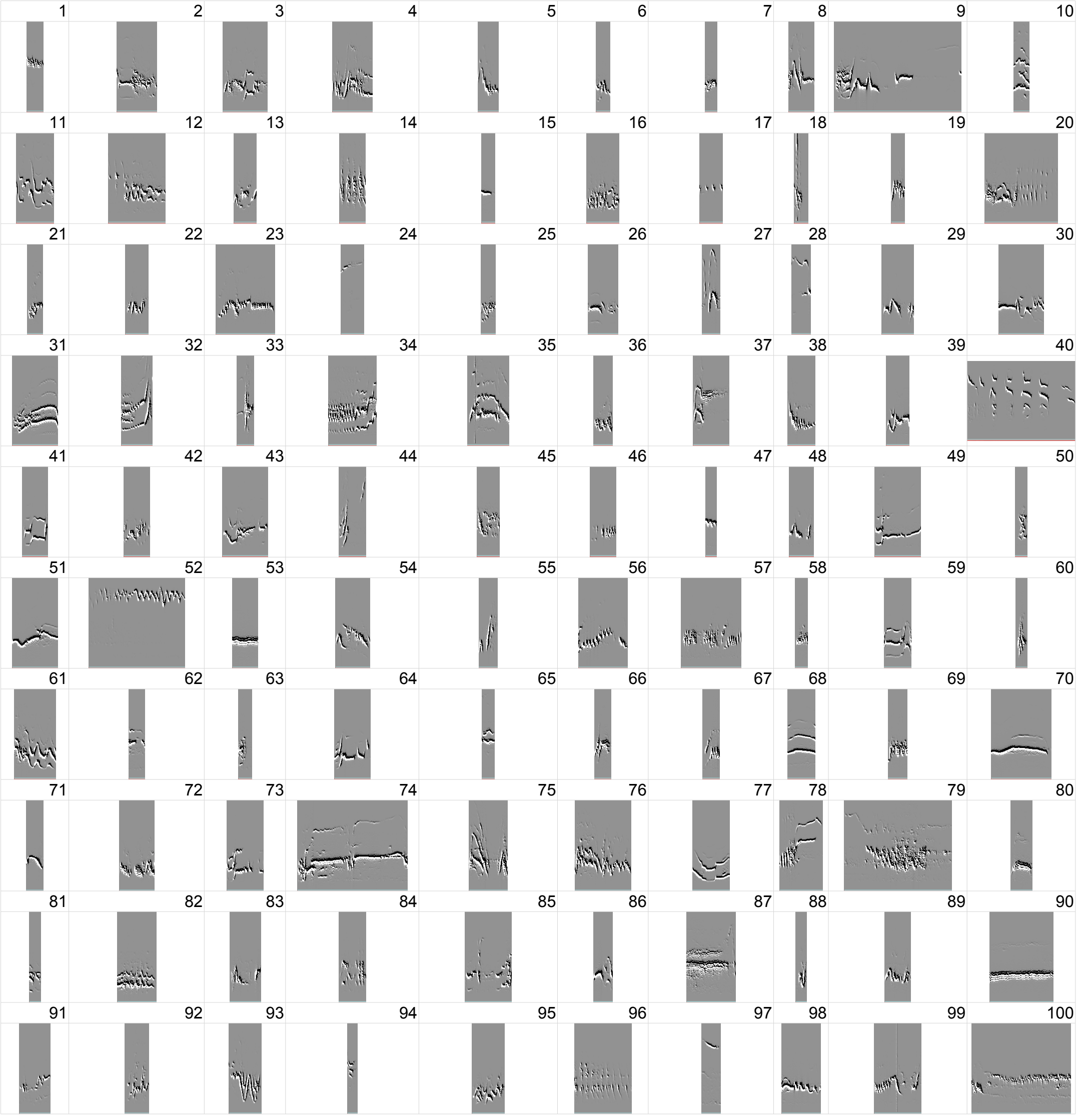

Supplement: Information S1 — Detailed description of California Thrasher song and of a method of phrase classification. (PDF) [file pone.0044436.s004.pdf]
